# Innate and adaptive immune response in SARS-CoV-2 infection-Current perspectives

**DOI:** 10.3389/fimmu.2022.1053437

**Published:** 2022-11-22

**Authors:** Qiugang Zhu, Yan Xu, Ting Wang, Feiting Xie

**Affiliations:** ^1^ Department of Laboratory Medicine, Shangyu People’s Hospital of Shaoxing, Shaoxing, China; ^2^ Department of Respiratory Medicine, Shangyu People’s Hospital of Shaoxing, Shaoxing, China; ^3^ Department of Laboratory Medicine, Affiliated Hospital of Jiangsu University, Zhenjiang, China; ^4^ Zhejiang Provincial Key Laboratory of Precision Diagnosis and Therapy for Major Gynecological Diseases, Women’s Hospital, Zhejiang University School of Medicine, Hangzhou, China

**Keywords:** COVID-19, SARS-CoV-2, immune responses, vaccination, treatment

## Abstract

Coronavirus disease 2019 (COVID-19) has been a global pandemic, caused by a novel coronavirus strain with strong infectivity, severe acute respiratory syndrome coronavirus 2 (SARS-CoV-2). With the in-depth research, the close relationship between COVID-19 and immune system has been dug out. During the infection, macrophages, dendritic cells, natural killer cells, CD8^+^ T cells, Th1, Th17, Tfh cells and effector B cells are all involved in the anti-SARS-CoV-2 responses, however, the dysfunctional immune responses will ultimately lead to the excessive inflammation, acute lung injury, even other organ failure. Thus, a detailed understanding of pertinent immune response during COVID-19 will provide insights in predicting disease outcomes and developing appropriate therapeutic approaches. In this review, we mainly clarify the role of immune cells in COVID-19 and the target-vaccine development and treatment.

## 1 Introduction

In December 2019, severe acute respiratory syndrome coronavirus 2 (SARS-CoV-2) emerged in Wuhan, China, a novel coronavirus that has never been reported before.The disease caused by SARS-CoV-2 is known as coronavirus disease 2019 (COVID-19), has spread rapidly in China and all over the world due to the high transmissibility, threatening human health and public safety (1). Clinical symptoms that patients exhibit include pyrexia, fatigue, cough, and even lead to organ function damage (2). Over the past years, the investigation of the interaction between the “attacker” and the “defender” has provided us more supporting evidences on host immune responses and the plan for therapeutic vaccines and drugs in the future. In this review, we mainly discuss the innate and the adaptive immune responses in COVID-19 to provide a deeper insight into the disease pathogenesis, thereby providing more information for COVID-19 treatments exploration.

## 2 Structure, infection and epidemiology of SARS-CoV-2

As a novel coronavirus, 79% and 50% genome sequence identity of SARS-CoV-2 are similar to SARS-CoV and Middle East respiratory syndrome coronavirus (MERS-CoV), respectively (3). There are six functional open reading frames in SARS-CoV-2, including ORF1a/ORF1b, spike (S), envelope (E), membrane (M) and nucleocapsid (N) from 5’ to 3’ in the genome (4). Up to now, real-time reverse transcriptase-polymerase chain reaction (RT-PCR) assays for SARS-CoV-2 nucleic acid mainly targeting ORF1a/1b, N, E and S genes are considered as the gold standard for confirming SARS-CoV-2 infection in patients (5). In addition, the amino acid identity of structural and non-structural proteins encoded by SARS-CoV-2 is similar to SARS-CoV. Apart from spike, another three structural proteins (envelope-membrane-nucleocapsid) have more than 90% amino acid identity with SARS-CoV, and most non-structural proteins are above 85% (3, 6).

The spike (S)-glycoprotein contains two functional domains, the amino (N)-terminal and carboxyl (C)-terminal named S1 subunit and S2 subunit, respectively (7). S1 subunit includes a receptor-binding domain (RBD), which is a key site in mediating the fusion of SARS-CoV-2 and host cell membrane during the infection. Being different from S1 subunit, the S2 subunit is a coiled helix structurethat contains a hydrophobic fusion loop and two heptad repeat regions. Previous reports have revealed these two domains exist as homo-trimer spikes and bind to the human Angiotensin-converting enzyme 2 (ACE2) host cell receptor in an accessible conformation *via* RBD domain of S1 subunit (8).

ACE2, a well-known receptor of SAR-CoV, has also been revealed to be the receptor of S-glycoprotein in SARS-CoV-2. ACE2 is widely expressed in human, bats, pangolins, pigs, ferrets, rhesus monkeys, civets, cats and dogs, which implies a wide host range of SARS-CoV-2 (9). For humans, the invasion of SARS-CoV-2 was not determined solely by ACE2 expression but also the assist by other factors (such as host proteases). For instance, the expression of ACE2 was limited in the respiratory tract which were favored by SARS-CoV-2 (10).

**Figure 1:**
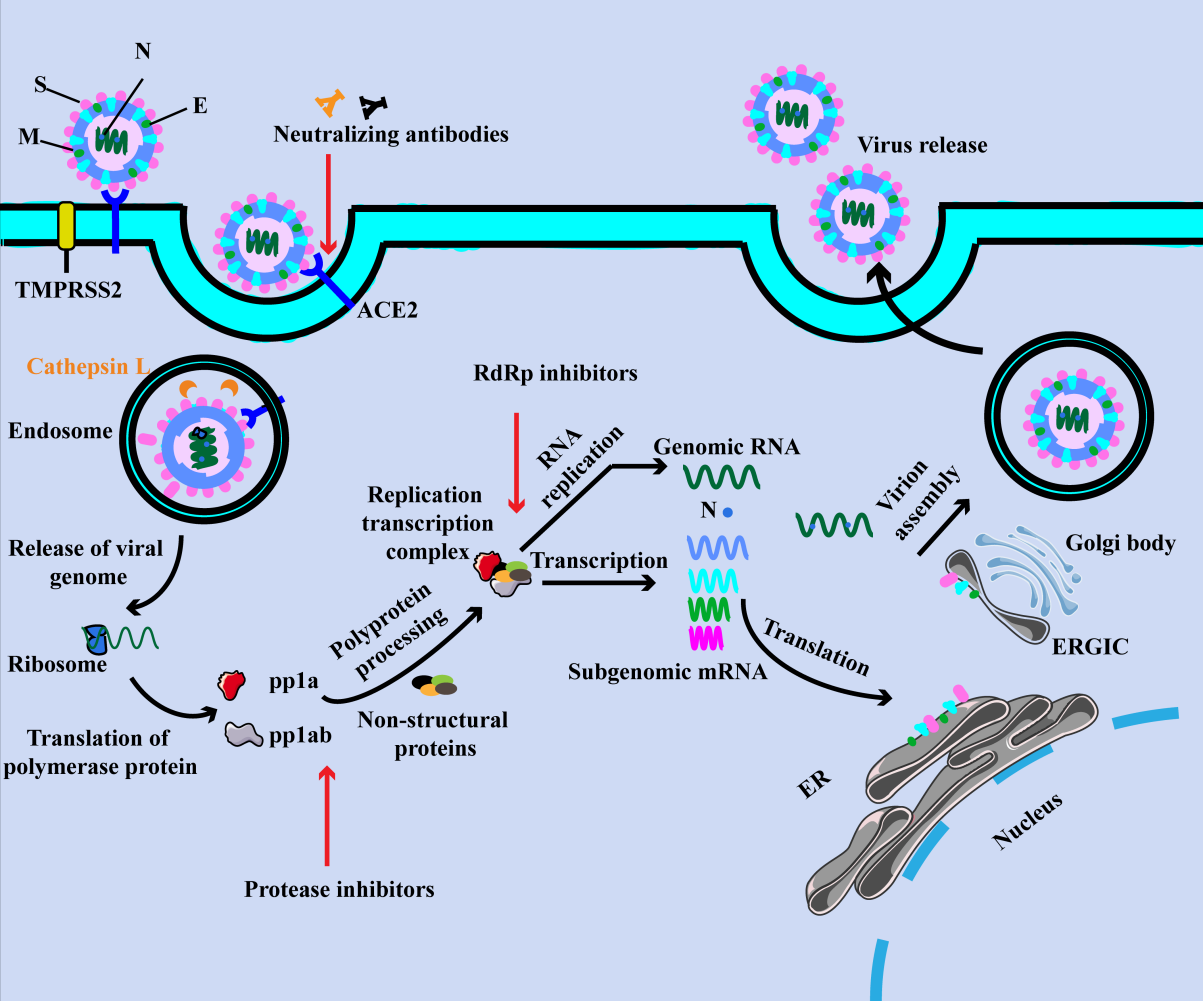
The SARS-CoV-2 virion and life cycle. The SARS-CoV-2 virion contains four structural proteins, namely spike (S), envelope (E), membrane (M), and nucleocapsid (N). The positive-sense, single-stranded RNA genome (+ssRNA) is encapsidated by N, whereas M and E ensure its incorporation into the viral particle during the assembly process. The spike (S) protein of SARS-CoV-2 specifically binds to ACE2, together with host factors (such as the cell surface serine protease TMPRSS2), facilitating the uptake and fusion of the virus in the cell or endosomal membrane. Following entry, the release and uncoating of the incoming genomic RNA allow it to the immediate translation of open reading frames. The resulting polyproteins pp1a and pp1ab are co-translated and post-translationally processed into the individual non-structural proteins (NSPs) that form the viral replication and transcription complex. Translated structural proteins translocate into endoplasmic reticulum (ER) membranes and transit through the ER-to-Golgi intermediate compartment (ERGIC), where they interact with N-encapsidated, newly produced genomic RNA and then lead tobudding into the lumen of secretory vesicular compartments. Finally, SARS-CoV-2 are secreted from the infected cell by exocytosis.

In the initial stage of infection, S1 protein binds to ACE2 *via* the RBD and then S1 protein shed from the virus surface, prompting the S2 domain membrane fusion with host cell (**Figure 1**) (11, 12). This process must be activated by cleavage of the S1 protein by the host protease to activate the S protein, including transmembrane protease serine protease 2 (TMPRSS2), cathepsin L and furin (11, 13). The study suggested that TMPRSS2 could be hired for the priming of SARS-CoV-2 S protein, which contributed to the fusion of the virus with the host cell membrane (13). Also, it could cooperate with cathepsin L to generate the cumulative effects in the fusion process (11). Additionally, other membrane proteins have also been confirmed to involve in this process. Interestingly, researchers from Peking University found that LDLRAD3, TMEM30A and CLEC4G effectively mediated virus invasion into cells in an ACE2-independent manner (14).

Similar to other coronaviruses, variants of SARS-CoV-2 have emerged in the past few years, and the main prevalent variant in the world today is Omicron variant (15–17). Variants might influence the epidemiological characteristics and clinical symptoms of COVID-19, even reducing the protection established by neutralizing antibodies and vaccines. Therefore, comprehending the immune responses to SARS-CoV-2 is of great importance for future vaccination and therapy.

Age is one of the main factors affecting the COVID-19 severity. Previous studies showed that every age group was susceptible to SARS-CoV-2, and in contrast to children (0-14 years old), old adults (above 65 years old), especially those with underlying diseases, are prone to develop severe respiratory diseases or even die (2, 18). Notably, some studies reported that SARS-CoV-2 could be transmitted to the fetus from pregnant women with COVID-19 through the placenta (19–21). Thus, all population are susceptible to SARS-CoV-2 through different ways.

## 3 Immune responses in COVID-19

**Figure 2:**
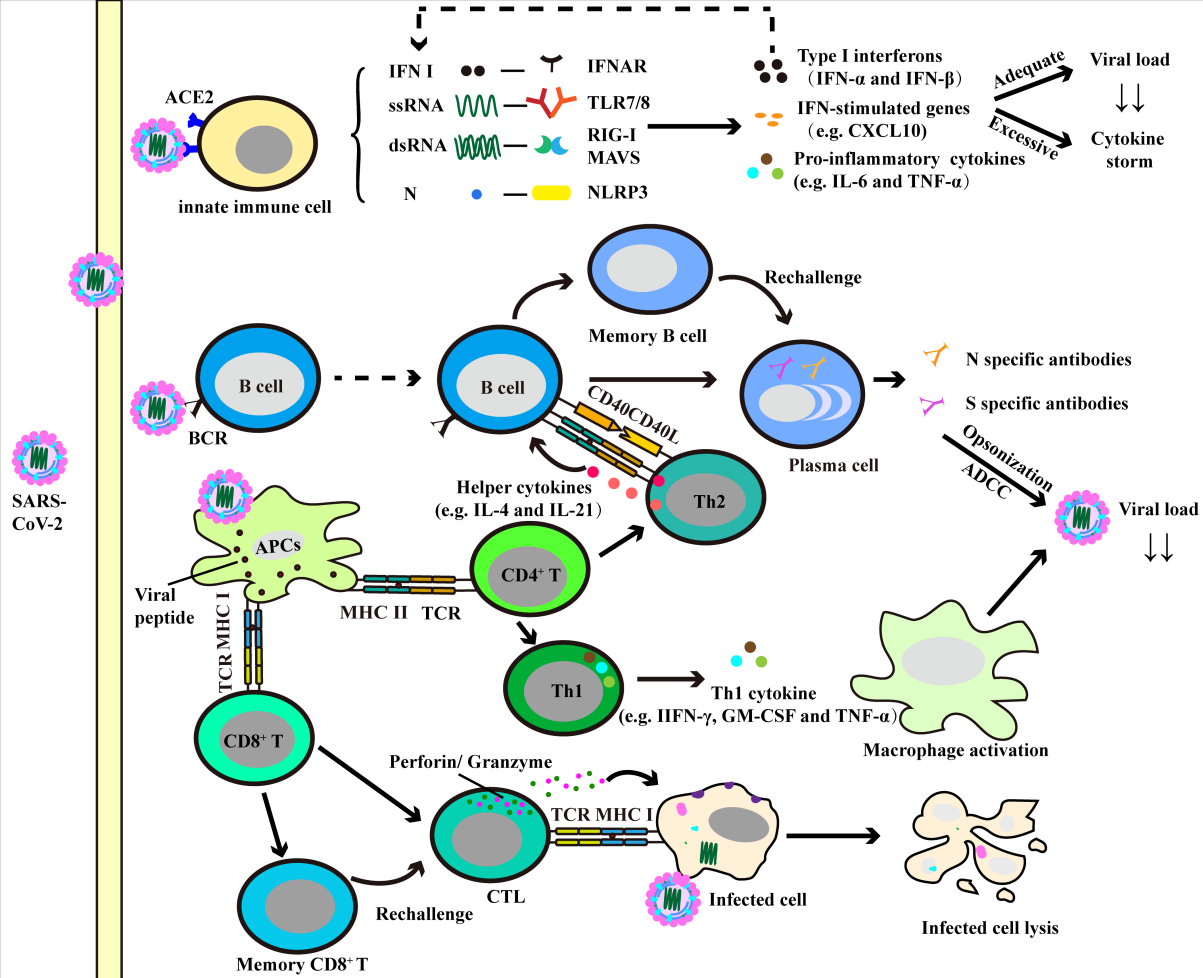
Immune response against SARS-CoV-2. The induction of innate immune cells (e.g. neutrophils, monocytes/macrophages and dendritic cells) results in production of various pro-inflammatory cytokines and chemokines. And the adequate production of these factors helps to clear the virus, while over-production can lead to cytokine storm and lung injury. The presentation of antigen by antigen-presenting cells (APCs) can induce humoral and cellular immunity in the human body. Specific CD4^+^ T cells can be activated and differentiate into Th cells, where Th1 cells promote the activation of macrophage-dependent virus clearance *via* producing macrophage activating factors (e.g. TNF-α and GM-CSF), and Th2 cells facilitate the humoral immunity. With the help of Th2 cells, B cells produce virus specific antibodies and neutralize viruses. CD8^+^ T cells differentiate into cytotoxic T lymphocyte (CTL), contributing to virus clearance by lysis of infected cells.

Along with the deepening of research, the role of immune cells in the anti-SARS-CoV-2 response has increasingly being recognized. In the following description, we will discuss both innate and adaptive immune responses in COVID-19 (**Figure 2** and **Table 1**).

**Table 1:** Roles of immune cells in the pathogenesis of SARS-CoV-2 infection.

Cell	Roles in viral progression	Function	References
Monocyte/Macrophage	Not duplicate	Cell pyrosis and induced profibrotic effects	(22)
pDC	Inhibiting	Mediated by IFN-I	(23)
cDC	Inhibiting	Mediated by IFN-I	(24)
NK cell	Inhibiting	Mediated by IFN-γ and TNF-α	(25)
	Antifibrotic activity	Inhibited expression of profibrotic genes in lung fibroblasts	
Th1 cell	Inhibiting	Mediated by IFN-γ	(26)
senescent Th2 cell	–	independent and significant risk factors for death	(27)
Tfh cell	Inhibiting	Supported GC responses	(28)
Tfr cell	Promoting	Suppressed anti-viral responses	(29)
Treg cell	Promoting	Suppressed the anti-viral responses *via* TGF-β and IL-10	(30)
CTL cell	Inhibiting	Secreted cytotoxic molecules	(31)
Effector B cell	Inhibiting	Secreted nAb against virus	(32–34)
Regulatory B cell	Promoting	Need further exploration	(35)

### 3.1 Innate immune responses in COVID-19

#### 3.1.1 Monocyte/Macrophage

Macrophages are myeloid immune cells that are involve in inflammation and tissue reparation. In lung, macrophages are divided into alveolar macrophages and interstitial macrophages. The former are mainly distributed near type I/II epithelial alveolar cells, while the latter preferentially reside between the microvascular endothelium and alveolar epithelium zone (36, 37). When pathogens and heterologous materials enter into lung, actived macrophages serve as the first line to resist the attackers. It is worth noting that short-lived macrophages can limit the replication of the virus, and once converted into long-lived macrophages, they might become infected resident cells (38).

Upon SASR-CoV-2 infection, the pro-inflammatory activity of macrophages was enhanced, as indicated by the increased expression of inflammatory cytokines (IL-1β, IL-6) and chemokines (CXCL10) by macrophages (39, 40). Macrophages adjacent to areas of endothelial cell damage were the main producers of IFN-I, which might be elicited by the stimulator of interferon genes (STING) induced-IFN-I production and mtDNA release from adjacent endothelial cells (41). Increased pro-inflammatory factors may led to the occurrence of cytokine storm, which was correlated with the poor prognosis of COVID-19 patients (42, 43). Independent infection experiments in human macrophages also corroborated that the induction of IFN-I, TNF, IL-1β and IL-6 generally resulted in IFN-I-mediated cell death (39). Studies have shown that S-protein in SARS-CoV-2 could elicit inflammasome formation and IL-1β production in macrophages from COVID-19 patients, and further exploration revealed that this process needed non-specific activation of monocytes *in vivo* to trigger NLRP3 inflammatory signaling (40). Macrophages express ACE2, furin and TMPRSS2, whether the three have a synergistic effect in the process of virus infection still need to be explored (44–46). SARS-CoV-2 infection might promote macrophages to adopt a profibrotic phenotype and result in lung fibrosis (22). *In vitro*, SARS-CoV-2 induced profibrotic programs in classical monocytes, as indicated by a profibrotic proteome profile that was also expressed in idiopathic pulmonary fibrosis (22). There were several articles looking at the future of mesenchymal stem cells (MSC) and MSC-derived extracellular vesicles (MSC-EVs) as living anti-inflammatory therapy for COVID-19, including inhalation of MSC-EVs that promoted the macrophages polarization into a M2 phenotype (47, 48). A novel ACE2-overexpressing microparticles (AO-MPs) could adsorb more virus and then deliver them into alveolar macrophages, consequently quenched in alveolar macrophages and ameliorated macrophage-mediated inflammation (49). Accordingly, macrophages are non-negligible players in SARS-CoV-2-elicited inflammation and lung firbrosis, and macrophages might be a target for COVID-19 treatment.

#### 3.1.2 Dendritic cell

A recent study revealed that patients with COVID-19 exhibited a defective DC response (24). In severe COVID-19 cases, plasmacytoid DCs (pDCs, critical effectors of antiviral immunity) exhibited increased pro-apoptotic pathways than those in moderate cases, accompanied by the downregulation of antiviral interferon-stimulated genes (ISGs) and innate sensors, TLR9, IFNAR and DHX36 (23, 24). pDCs are one of the main producers of IFN-I, which might produce more IFN-I than other immune cells or infected cells (50). Infiltration of pDCs (sensing SARS-CoV-2) were positively correlated with IFN-I production, contributing to the macrophage-mediated cytokine storm in the lungs of COVID-19 patients and might increase the lung inflammation (51). In detail, IFN-α production following TLR7 sensing of SARS-CoV-2 in pDCs mediated the transcriptional and epigenetic changes in macrophages, which facilitated their hyperactivation upon environmental stimuli (51). Comparing with moderate cases, conventional DCs (cDCs) from patients with severe COVID-19 exhibited decreased IFN signature and MHC II expression, contrary to pro-inflammatory pathways (24). Further exploration suggested that this reduction was correlated with low expression of MHC-II regulators (RFX5, RFXANK and CIITA) in severe cases. This viral inhibition of antigen presentation may explain the disease aggravation in severe cases and provide novel targets to restore the defective antiviral defense (24). Above all, DCs may have the potential to be a target to enhance the protective effects triggered by vaccines.

#### 3.1.3 Natural killer cell

NK cells from healthy individuals showed anti-viral activity, as indicated by the reduced SARS-CoV-2 protein levels in cocultured infected cells (52). In addition to the anti-viral activity, NK cells also exhibited antifibrotic activity by the decreased expression of profibrotic genes COL1A1 and ACTA2 in lung fibroblasts (25). In contrast to the healthy individuals, NK cells from patients with COVID-19 showed limited anti-viral activity with an exhausted phenotype (53, 54). Previous studies have reported that the quantity of NK cells was significantly reduced in COVID-19 patients, displaying an exhausted phenotype by increased expression of NK inhibitory marker NKG2A (53, 55). Nevertheless, increased expression of NKG2A led to the reduced levels of CD107a (degranulation marker), IFN-γ, IL-2, granzyme B, and TNF-α in NK cells from COVID-19 patients (53). COVID-19-associated NK cells display a genetic profile similar to that of pulmonary NK cells from patients with pulmonary fibrosis, accompanied by the decreased ability to suppress the profibrotic gene expression (mentioned above) in lung fibroblasts (25). As an important anti-viral effector molecule, IFN-α suppressed the IFN-γ production by NK cells, but neutralization of IFN-α was unable to rescue the function of NK cells.

Based on the reduced anti-viral activity and antifibrotic activity of NK cells in patients with COVID-19, recovering their activities might be promising therapies against SAR-CoV-2. A recent study revealed that BNT162b1 mRNA vaccine significantly increased the quantity of CD56^bright^, CD56^dim^, and CD56^dim^/CD16^dim^ NK cells, and with an increase in IFN-γ, perforin and granzyme content (56). This result offered further support to end the SARS-CoV-2 pandemic with worldwide vaccination efforts (56). Therefore, the benefits of NK cell-based immunotherapy in patients with COVID-19 require further study.

Immunity against viral infection mainly begins with the recognition by pattern recognition receptor (PRRs) that are widely distributed on a variety of immune and non-immune cells. Signaling *via* PRRs induce the production of type I/III IFNs and pro-inflammatory cytokines, which trigger the transcription of ISGs to restrict viral infection. Valuable descriptive and correlative information about COVID-19 and innate immune responses (monocytes/macrophages, dendritic cells and NK cells) have been gathered during the pandemic. In addition to the above discussion, other innate immune cells (including neutrophils, eosinophils and epithelial cells) also have an important role in COVID-19 prediction and development. For example, neutrophils responses and neutrophil extracellular traps (NETs, contain nuclear component, microbicidal proteins and oxidative enzymes derived from neutrophils to limit infection) are correlated with the hyperinflammation in COVID-19 patients (57). Thus, targeting innate immunity may become a new strategy for the treatment of COVID-19.

### 3.2 Adaptive responses to SARS-CoV-2

Virus-specific cellular and humoral responses are the main weapons in protecting patients after SARS-CoV-2 infection. Next, we will comprehensively discuss the role of adaptive immunity in the fight against SARS-CoV-2.

#### 3.2.1 CD4^+^ T cell

Different CD4^+^ T cell subsets can play beneficial or detrimental roles in the progression of COVID-19. Highly activated, functional CD4^+^ T cells and functionally exhausted CD4^+^ T cells have been detected simultaneously in individuals with severe COVID-19 (58). Previous studies have demonstrated that 83% of CD4^+^ T cells in patients with COVID-19 were S reactive, which could respond to both N and C terminal of S protein (59). Thus, the interest in studying S protein-specific antiviral immune responses (including T-cell responsive COVID-19 vaccine) has been stimulated (60, 61). SARS-CoV-2-specific CD4^+^ T cells were associated with ameliorated COVID-19 disease severity, and the induction of SARS-CoV-2-specific CD4^+^ T cells was correlated with rapid decrease of viral load (62, 63). Another study also confirmed that early viral clearance and less critical illness were correlated with the higher counts of CD4^+^ T cells, particularly naïve CD4^+^ T cells (64).

##### 3.2.1.1 Th1, Th2 and Th 17 cells

The disproportionality of T helper type 1 (Th1) or Th2 cells has been well elucidated in patients suffered from COVID-19 pandemic and non-infected people who are at maximum risk of infection (26). In an observational study,COVID-19 patients have been reported to have lower proportion of Th1 and Th17 cells and a higher proportion of activated Th2 cells compared with control population (27). In addition, higher proportion of senescent (PD1^+^/ICOS^-^) Th2 cells in dead patients than in survivors (27). By evaluating the relationship between Th subsets and mortality, they found that the total lymphocytes and the higher percentages of senescent Th2 cells seem to be independent and significant risk factors for death (27). In the stage of recovery, the proportion of both Th1 and Th2 cells in patients were decreased than in healthy controls (27, 65). Thus, the dynamic change of Th cells might be a tool to monitor the development of COVID-19.

Th17 cells play pivotal roles in tissue damage and excessive inflammation *via* IL-17 and IL-23 (66). Researchers have found that high levels of Th17 cells in the periphery of patients infected with SARS-CoV-2 (30, 67, 68). A new study revealed that IL-17A expression was significantly associated with the severity of the disease (69). The results of studies also suggested that a systemic neutrophil environment may preferentially skew CD4^+^ T cells toward Th17 and IL-17A promotion, thereby exacerbating inflammation (69, 70). Another study’s results were consistent with this phenomenon, further experiment provided the evidence that this Th17 promotion could be reversed by inhibitors of arginase-1 (Arg-1), NO synthase (NOS), and reactive oxygen species (ROS), all produced by myeloid cells under oxidative stress (71). Thus, for clinical benefit, targeting Th17 promotion and blocking IL-17 signaling to manage COVID-19 patients, particularly those presenting with cytokine storm syndrome is promising.

##### 3.2.1.2 Tfh cells

Among CD4^+^ Th cell subsets, follicular helper T (Tfh) cells participate in humoral immunity *via* interacting with B cells in the germinal center (GC), facilitating the differentiation of B cells into plasma cells to product high-affinity neutralizing antibodies (28). Researchers revealed the absence of GC in thoracic lymph nodes and spleens from COVID-19 patients, accompanied by the reduction of Bcl-6^+^ GC B cells in both organs (72). The reduction of CD4^+^CXCR5^+^Bcl-6^+^ germinal center Tfh cells might explain the loss of GCs and the accumulation of non-GC-derived activated B cells (72). However, SARS-CoV-2 infection increased the number of peripheral CD4^+^ Tfh and GC responses in rhesus macaques (73). In clinical studies, convalescent individuals who experienced severe COVID-19 showed a higher induction of CXCR3^+^ Tfh cells when compared with those non-severe COVID-19-convalescent individuals. And the frequencies of them were positively associated with neutralizing antibody titers in convalescent individuals (74). In addition, they intriguingly found that circulating Tfh cells were S-protein specific and functional in spleen (73, 74). Moreover, researchers found that circulating S-specific Tfh cells could persist in constant numbers in the blood and lymph nodes for more than six months (75). In this study, they highlighted the importance of virus-specific Tfh cell responses in mRNA vaccine-induced robust and durable immune protection against SARS-CoV-2 (75). Furthermore, in Tfh-deleted mice immunized with S protein vaccination, Tfh cells also played an indispensable role for the formation of optimal SARS-CoV-2 Spike-specific B cell in GCs and for somatic hypermutation (SHM) (76).

In addition to the above protective effects, Tfh cells could also accelerate the disease progression in certain cases. Compared to non-hospitalized COVID-19 patients, severe cases showed increased cytotoxic Tfh cells which manifested high levels of IFN-γ, IL-2, and TNF-α (77). In an observational study, the results showed that CCR4^+^ and CCR6^+^ Tfh cells could be pathogenic in COVID-19 patients (78). In addition, attention needs to be focused on COVID-19 patients those who have Tfh-associated diseases, such as HIV, autoimmune diseases, who are more likely to develop severe disease (79, 80).

##### 3.2.1.3 Treg cells

Regulatory T cells (Tregs) keep the immune homeostasis *via* multiple mechanisms thus the role of Tregs has not allow to ignore in these processes (81). Flow cytometric analysis showed an increased proportion of Tregs in peripheral blood mononuclear cells (PBMCs) with enhanced expression of FoxP3, which correlated with poor prognosis (82). In mechanically ventilated patients with COVID-19 acute respiratory distress syndrome (ARDS, hypoxemic respiratory failure caused by inflammation within the lung), in sharp contrast to the lymphopenia in both subsets of CD4^+^ and CD8^+^ T cells, the proportions of Tregs were increased in the lungs and PBMCs (83). The recruitment of Tregs into the lungs of COVID-19 patients may determine the severity of the disease since patients with more Treg cells experienced the milder disease (84). However, recent clinical studies showed that CD3^+^CD4^+^CD25^hi^CD127^lo^FoxP3^+^ Tregs in PBMCs markedly reduced in severe COVID-19 patients (77, 85, 86). In ICU patients with COVID-19, the quantity of Tregs in PBMCs and their functional molecules (TGF-β and IL-10) were significantly reduced, accompanied by an increased ratio of Th17/Treg cells, RORγt/FoxP3, and IL‐17/IL‐10 in patients compared with the controls (30). In adults and children with severe COVID-19, the proportion of Tregs in CD4^+^ T was also decreased (87). When recovered from mild COVID-19, patients seem to exhibit decreased levels of Tregs compared to themselves during hospitalization, and severe convalescents resulted in perturbances in CD4^+^ Tregs (88, 89). Moreover, Treg-targeted therapies have been proposed and might be promising, including the adoptive transfer of Tregs and rIL-2 administration (NCT04468971 and NCT04357444, ClinicalTrials.gov) (90). Above all, a comprehensive understanding of the roles of Treg in COVID-19, and identification of the mechanisms regulating the balance between Tregs and other Th subsets may offer therapeutic novelties for COVID-19 infection.

#### 3.2.2 CD8^+^ T cells

It was reported that SARS-CoV-2-specific CD8^+^ T-cell responses might be associated with disease severity during the acute phase, and decreased number has been observed in severe cases (31, 62, 91). For example, a study suggested that weak CD8^+^ T cell responses, despite high antibody titers, might contribute to the pathogenesis of acute COVID-19 (54). Depletion of CD8^+^ T cells in vaccinated macaques prior to SARS-CoV-2 infection led to the higher levels of peak viral load and day 4 in both the upper and lower respiratory tracts (92). CD8^+^ T cells from COVID-19 patients exerted enhanced production of IL-2, IL-17, and the expression of degranulation marker CD107a upon anti-CD3/CD28 stimulation when compared to cells from healthy controls (70). In contrast to the previous conclusion, several studies reported that CD8^+^ T cells from COVID-19 patients exhibited a decreased cytokine-producing capacity (53, 91). However, all this information was not based on SARS-CoV-2 antigen stimulation. There was increasing evidence that circulating CD8^+^ T cells from severe COVID-19 patients with exhibited an exhausted phenotype characterized by increased expression of PD-1, TIM-3, LAG-3, CTLA-4, NKG2A, and CD39, and there was an inverse relationship between the expression of PD-1 and CD38 (an activation marker) in CD8^+^ T cells (53, 93–96). Accumulated evidence revealed that the expression of PD-1 in CD8^+^ T cells from COVID-19 patients was increased, especially ICU patients (95). Moreover, a recent study revealed that SARS-Cov-2-specific CD8^+^PD-1^+^ T cells produced more IFN-γ than PD-1-negative populations regardless of disease severity, indicating a functional state of SARS-CoV-2-specific CD8^+^PD-1^+^ T cells (96).

In the acute phase of COVID-19, SARS-CoV-2-specific CD8^+^ T cells expressed activation markers (CD38 and HLA-DR) and secreted cytotoxic molecules (perforins and granzyme B), indicating these cells were activated with cytotoxic functions (31). Nasal-resident SARS-CoV-2-specific CD8^+^ cells could be detected almost exclusively in vaccinees who experienced SARS-CoV-2 breakthrough infection (persisted for ≥140d) (12). And these CD8^+^ T cells were predominantly tissue-resident memory T cells which had an elevated proportion of activated cells (97, 98). Moreover, researchers have found that memory CD8^+^ T cells were required for the clearance of SARS-CoV-2, which was reflected by a delayed decreased of viral loads in the respiratory tract in CD8^+^ T cells-depleted and SARS-CoV-2 rechallenged convalescent rhesus macaques (99). It’s worth noting that memory CD8^+^ T cells found in a study of UK convalescent individuals following COVID-19 would support an understanding of protective immunity and may contribute to put forward potential immune-based therapeutics (100). Vaccine-induced CD8^+^ T cells also help to fight against SARS-CoV-2 re-infection (101–103). One week after prime vaccination with BNT162b2, a stable and fully functional CD8^+^ T cell response was mobilized, as indicated by the increased production of IFN-γ and TNF-α (104). In addition, boost vaccination-induced highly differentiated effector CD8^+^ T cells and vaccination-induced early memory T cells exhibited similar capacities (104).

### 3.3 B cells

The alterations of B cell subpopulations in COVID-19 have been reported, either immature or terminally differentiated. Compared to healthy subjects, the frequencies of CD19^+^ B cells in PBMCs seem to be slightly increased in severe and critical cases (105). The double negative (DN) fraction (CD27^-^IgD^-^) is one of B cell compartments that showed significant alterations in COVID-19 (105). Depending on the disease severity, mean frequencies of DN1 (CD21^+^CD11c^-^), DN2 (CD21^-^CD11c^+^), and DN3 (CD21^-^CD11c^-^) subsets exhibited shifted distributions. DN1 B cells showed a significant reduction in severe and critical cases. DN2 B cells displayed a significant increase in severe cases vs mild/moderate and critical cases while DN3 B cells were significantly increased as disease severity increased. In addition, immature, activated naïve B cells and the DN3 fraction showed a strong correlation with ventilatory parameters, such as respiratory rate, SpO2, and PaO2/FiO2 (105). Another study also revealed similar phenomenon about DN2 B cells in acute patients with COVID-19 (106). This study also revealed that severe patients displayed enhanced extrafollicular B cell activation with accelerated inflammation, while mild patients counteracted the disease through the timely induction of mitochondrial dysfunction in B cells, which suppressed the extrafollicular responses, resulting in increased neutralizing potency index and reduced inflammation (106).

Germinal centers (GCs) are transient microstructures formed within the follicles of secondary lymphoid tissues. Within GCs, activated B cells undergo clonal expansion and affinity maturation and get further help from Tfh cells to differentiate into memory B cells or long-lived plasma cells, which is of vital importance for the generation of humoral immunity (107). In severe SARS-CoV-2 infection, GC responses were suppressed, which inhibited the production of neutralizing antibody (nAb). Multicolor histological assessments revealed that GCs were also largely absent in thoracic lymph nodes and spleens from patients succumbing to COVID-19. There were also the absence of BCL6^+^ B cells or Tfh cells, both were the contributors to GCs (72). Furthermore, they also speculated that excess TNF-α inhibited the formation of GC responses in COVID-19 *via* blocking Tfh cell differentiation (72, 108, 109).

Following infection with SARS-CoV-2, naïve B cells and existed memory B cells (upon early exposure of other coronaviruses) will be activated and differentiate into antibody-secreting cells (ASCs). Discrete increments of ASCs were seen in patients with mild/moderate COVID-19 and were significantly amplified with disease severity (105). In contrast to the majority infection, the serum IgG appeared at approximately the same time as serum IgM and IgA responses to SARS-CoV-2 S and N, usually within the first 2 weeks after symptom onset (32–34). Importantly, the memory B cell is the only B cell subset that correlated with a clinical outcome in hospitalization period (105). A recent study revealed that individuals recovered from COVID-19 developed SARS-CoV-2-specific immunoglobulin (IgG) antibodies, neutralizing plasma, and memory B and memory T cells which could persist for at least 3 months. Moreover, SARS-CoV-2-specific IgG memory B cells (MBCs) increased over time (110). S-RBD-specific class-switched MBCs were detected in 13 of 14 participants, failing only in the individual with the lowest plasma levels of anti-S-RBD IgG and neutralizing antibodies. Resting MBCs (rMBCs) made up the largest proportion of S-RBD-specific MBCs patients with mild disease and hospitalized patients with moderate to severe disease (111). Another study found that in mild and severe COVID-19 patients, serum neutralizing antibody (nAb) responses waned rapidly but spike (S)-specific IgG^+^ memory B cells (MBCs) remained stable or increased. Analysis of 1,213 monoclonal antibodies (mAbs) isolated from S-specific MBCs displayed increased somatic hypermutation, binding affinity, and neutralization potency over time, indicating prolonged protection *via* humoral immunity (111). A recent study introduced that the effect of MBCs elicited by mRNA vaccines in preventing SARS-CoV-2 variants (including Omicron variants), highlighting the protective roles of MBCs in disease (112).

The adaptive immune system is important for control of most viral infections. We have discussed the roles of Th, Tfh, CD8^+^ T and B cells in the pathogenesis of COVID-19, and other adaptive immune cells, such as Tfr cells, also contribute to optimize humoral immunity, meaning their importance to avoid the overactivation of immune responses. Whether viral-inhibiting or viral-promoting effects of immune cells in COVID-19, these researches state the importance of them in anti-SARS-CoV-2 and define emergence of new paradigms in anti-viral activity.

## 4 Vaccine development

Vaccination is considered as one of the greatest successes in the history of medicine, it could decrease the disease incidence, or at least suppress its detrimental or even fatal clinical manifestations. At present, there are mainly five kinds of SARS-CoV-2 vaccines, including inactivated vaccines, recombinant protein vaccines, viral vector-based vaccines, live attenuated vaccines and nucleic acid vaccines (113). As the first inactivated SARS-CoV-2 vaccine, PiCoVacc induced SARS-CoV-2-specific neutralizing antibodies in mice, rats and non-human primates, and these antibodies showed a broader neutralizing ability against other 10 representative SARS-CoV-2 strains (114). *In vivo*, vaccinated macaques related symptom ameliorated, as indicated by the mild and focal histopathological changes and viral load compared with the controls (114). In phase 1/2 clinical trials, CoronaVac has been confirmed to be safe and well-tolerated, inducing humoral responses in children and adolescents aged 3-17 years and healthy adults (≥18 years old) (115–117). The phase 3 trial in Turkey also revealed a vaccine efficacy of 83.5% in the vaccine group with good safety and tolerability profile (118). In phase 4 studies, scientists found that low antibody concentrations 6 months after previous immunisation with two doses of CoronaVac. However, a third dose induced a significant increase of neutralizing antibodies, which could improve protection against infection. Importantly, heterologous boosting produce a more stronger immune response than homologous boosting (119). The BNT162b2 mRNA and mRNA-1273 COVID-19 vaccine are the most widely used mRNA vaccines, and both vaccines showed more than 90% protective efficiency (120, 121). Viral vector-based adenoviral vaccines have also been explored, including adenovirus-vectored vaccines. Exploration conducted by Chen’s team showed that the recombinant adenovirus type 5 transmitted COVID-19 vaccine was safe in a single dose, and induced robust immune responses in healthy adults aged 18 years or older and children or adolescents (122–124). Humoral responses peaked at day 28 post-vaccination in healthy adults, and rapid specific T-cell responses were noted from day 14 post-vaccination (122). Another adenovirus-vectored vaccine, ChAdOx1 nCoV-19 vaccine also exerted protective effective against SARS-CoV-2 (125, 126). Recently, recombinant tandem-repeat dimeric RBD-based protein subunit vaccine (such as ZF2001) also have been investigated and showed a good protective effect (127, 128). Scientists evaluated the SARS-CoV-2-spike-specific immune responses to Moderna mRNA-1273, Pfizer/BioNTech BNT162b2, Janssen Ad26.COV2.S, and Novavax NVX-CoV2373 (129). 100% of individuals made memory CD4^+^ T cells, and cTfh and CD4-CTL highly represented only after mRNA or NVX-CoV2373 vaccination, and a differentiating feature of Ad26.COV2.S immunization was a high frequency of CXCR3^+^ memory B cells (129). mRNA vaccinees had substantial declines in antibodies, while memory T and B cells were comparatively stable (129). A recent study demonstrated that Omicron infection enhances pre-existing immunity elicited by vaccines and, higher neutralization titres against all variants of concern (VOC) (130). Thus, Omicron infection may confer broad protection against non-Omicron variants in vaccinated individuals (130). Although there are different variants (Beta, Delta, Omicron and others), these vaccines are still effective especially in preventing the occurrence of severe cases (131–134).

Although vaccines can prevent the occurrence of severe diseases, accumulated evidence still showed that additional protective measures should be maintained to fight against variants with increased infectivity (135–137). In particular, as time goes on, the vaccine induced immune response will naturally decline.

## 5 Potential treatment of COVID-19

Currently, COVID-19 pandemic is one of the greatest threats to human health with more than 611 million confirmed cases and over 6.51 million deaths reported worldwide (as of September 23, 2022, sources from WHO). Various drugs were proclaimed to be effective against SARS-CoV-2, such as remdesivir, paxlovid, lopinavir/ritonavir, molnupiravir, and some monoclonal antibodies (138).

Monoclonal antibodies are used as a potential therapeutic and preventive drug for viral infection due to its high specificity and the ability to enhance immune response. Bamlanivimab (LY-CoV555, IgG1), a potent anti-spike neutralizing antibody, block the binding and entry to human cells (139). For primary outcomes, no differences were observed in bamlanivimab vs placebo, but bamlanivimab treatment was associated with lower day 3 nasopharyngeal viral levels and faster reductions in inflammatory markers and viral decay (140). One of three doses of bamlanivimab appeared to promote the natural decline in viral load over time (141). Moreover, compared to the placebo group, patients within LY-CoV555 group had a lower ratio of COVID-19-related hospitalization or visit to an emergency department (141). When combined with another monoclonal antibody, Etesevimab, the incidence of COVID-19-related hospitalization and death was decreased than the placebo group, accompanied by the decline of viral load (142). Sotrovimab (IgG1), casirivimab (IgG1-κ) and imdevimab (IgG1-λ), as S protein RBD specific antibodies which could prevent the binding of SARS-CoV-2 to the human ACE-2 receptor, may contribute to COVID-19 patients recover (143–145). Tocilizumab, a monoclonal antibody binds specifically to both soluble and membrane-bound receptors for IL-6and has been explored in the treatment of COVID-19. The results showed that the mortality in the tocilizumab group was lower than not treated with tocilizumab (146).

Remdesivir, an adenosine analogue, can be metabolized to its active metabolite remdesivir triphosphatea (an analogue of ATP), and integrate into the nascent viral RNA *via* RNA polymerase, resulting in delayed chain termination during replication, thereby inhibiting viral replication (147). In 2020, a study reported that patients with severe COVID-19 receiving remdesivir had a numerically faster time to clinical improvement than those patients receiving placebo when the duration of symptoms was 10 days or less (148). The ratio of adverse events was similar in both groups, but the proportion of patients who stopped clinical trial was higher in remdesivir group (12%) than placebo group (5%) (148). A 5 day remdesivir therapy of COVID-19 patients showed greater clinical improvement than patients in placebo group, but not different from a 10 day therapy (149). For cases treated in the ICU, remdesivir use within 9 days from symptom onset reduced mortality risk compared with control group (150).

Janus activated kinase (JAK) inhibitors also have been applied to the treatment of COVID-19 (151, 152). Baricitinib is a selective inhibitor of JAK1 and JAK2, both of which mediate signaling for inflammatory cytokines involved in inflammation and the immune response of COVID-19 (151). The results indicated a lower case fatality rate within 2 weeks in the baricitinib group (0%) than the control (6.4%) (152). In addition, the ICU admission rate was lower in patients receiving baricitinib treatment (0.88%) than controls (17.9%) (152). Tofacitinib is another inhibitor of JAK, which has been shown to inhibit the activity of JAK1, JAK2, JAK3, and to a lesser extent of Tyk2 (153). The results of a clinical trial have demonstrated that tofacitinib led to a lower risk of death or respiratory failure through day 28 than placebo (18.1% vs 29.0%), implying the therapeutic effects of tofacitinib in COVID-19 (154). By day 28, death occurred in 2.8% of patients in the tofacitinib group and in 5.5% of patients in the placebo group (154). Thus, the use of JAK inhibitors in hospitalized patients with COVID-19 should be considered to improve the clinical outcome and prevent death.

Paxlovid is a therapeutic combination consisting of two compounds: nirmatrelvir and ritonavir (155). Ritonavir could inhibit the metabolism of nirmatrelvir and allow the administration of lower doses of this substance (156). A recent study revealed that among patients who received paxlovid treatment within three days of symptom onset, only 0.8% patients were admitted to hospital by day 28 after randomization and no deaths. In comparison, these two values in the control group were 7% and 7 (157). In participants treated within five days of symptom onset, the effect of paxlovid was still significant. In addition, no deaths were reported in the paxlovid group as compared to 10 deaths (1.6%) in the placebo group (157). During the pandemic of COVID-19 in Shanghai (2022), scientists found that unvaccinated, delayed use of paxlovid (started 5 days after diagnosis) and immunosuppressive status were independent risk factors for delayed virus clearance (158). Compared with patients who delayed paxlovid treatment, immunosuppressive patients who began to use paxlovid within 5 days after diagnosis had an earlier virus clearance time of about 6 days. Moreover, correlation analysis showed that the earlier paxlovid was used, the more rapidly the virus was cleared (158). Although the benefit of paxlovid has been confirmed and has been approved in some contries, the furture of paxlovid still need further exploration (159).

Molnupiravir is a new oral antiviral drug that has recently been tested in COVID-19 (160). The results of phase clinical trial (NCT04575597) of molnupiravir showed that it can significantly reduce the risk of hospitalization and mortality in mild to moderate COVID-19 patients. Compared with placebo, patients taking molnupiravir had a 50% lower risk of hospitalization or death. More impressively, no deaths were reported in the group of patients taking molnupiravir, while there were 8 deaths in the placebo group. In terms of side effects, there was no difference between the two groups (161–163). Also, molnupiravir accelerated SARS-CoV-2 RNA clearance and elimination of infectious virus in patients with COVID-19 (median 14 days vs 15 days, log rank p= 0.013) (164). In this study, molnupiravir was well tolerated across all doses (200/400/800 mg), and the adverse events were mainly low-drade that were similar to those reported by participants in placebo group (164). For SARS-CoV-2 variants, a recent study found that SARS-CoV-2 Omicron variant was highly sensitive to molnupiravir, nirmatrelvir, and the combination (165). These evidence suggest molnupiravir is a promising drug in treating COVID-19.

Azvudine, a nucleoside reverse transcriptase inhibitor that previously been used in HIV-1 infection (166). In recent years, the effects of azvudine in COVID-19 have been revealed (167, 168). A randomized, open-label, controlled clinical trial (ChiCTR2000029853, http://www.chictr.org.cn) contained 20 mild and common COVID-19 patients showed that the mean times of the first nucleic acid negative conversion (NANC) of azvudine group and the control group were 2.6 days and 5.6 days, respectively (p = 0.008) (167). Also, the mean time of the first NANC of newly diagnosed subjects in the azvudine group was shorter (167). Thus, azvudine is an effective anti-SARS-CoV-2 drug in treating COVID-19 patients, and it has been approved to treat COVID-19 in China (169).

Diamidobenzimidazole 4 (diABZI-4), an STING agonist, has been confirmed to induce a rapid short-lived activation of STING, then the transient expression of proinflammatory cytokines and lymphocyte activation (170). *In vitro*, diABZI-4 significantly inhibited SARS-CoV-2 replication in infected cells (170, 171). In mouse model, the results showed that the viral load in both nasal cavity and lung was lower than the control, consequently prevented the disease progression (171). For variants (B.1.351), diABZI-4 showed good antiviral effect as well (171). In this process, attention needs to be taken to avoid excessive production of pro-inflammatory cytokines and cytokine storm.

As an important part of world medicine, Chinese traditional medicine is safe and effective in the treatment of COVID-19. The results showed that after 14 days of treatment with Lianhua Qingwen capsule, the cure rate of main clinical symptoms (fever, fatigue and cough) in the treatment group was significantly higher than the control group, reaching 57.7% on the 7th day, 80.3% on the 10th day and 91.5% on the 14th day. The duration of single symptoms of fever, fatigue and cough was also shortened. In terms of reducing the proportion of patients with severe disease, the treatment group of Lianhua Qingwen capsule was significantly lower than the control group (Lianhua Qingwen treatment group: 2.1%, control group: 4.2%) (172). Another study using network pharmacological strategy revealed that AKT1 is a promising drug target to reduce tissue damage and help eliminate virus infection (173). Practically, Chinese traditional medicine have been written up in COVID-19 diagnosis and treatment plan in China (173).

## 6 Conclusion

The current knowledge about SASR-CoV-2 indicates that the immune system plays a crucial role in setting the severity of COVID-19. Although an extraordinary amount has been accomplished during the last three years, the relationship between immune responses and the severity of diseases still remains to be explored. Appropriate responses against SARS-CoV-2 could ameliorate the symptoms of COVID-19 and prevent the occurrence of severe diseases, while excessive responses elicited cytokine storm and pathogenic B cell activation, increasing the risk of death. Thus, in immune-based therapeutics, avoiding the excessive activation of immune cells will also be the proposition of our future research. However, understanding heterogeneous disease manifestations of COVID-19 remains a major knowledge gap, and exploring the relationships between COVID-19 and immunity is a priority. The emergence of new mutant strains has also brought unprecedented pressure to vaccine development. With the advent of new technologies such as next-generation sequencing (NGS), CRISPR-based assays and nanotechnology, enhanced the the accuracy and sensitivity of COVID-19 diagnosis and treatments. Furthermore, duration of immune memory and protective immunity to SARS-CoV-2 after infection or vaccination will be a high priority for years to come.

## Author contributions

QZ drafted the manuscript. YX and TW discussed and revised the manuscript. FX conceived the topic and revised the manuscript. All authors contributed to the article and approved the submitted version.

## Conflict of interest

The authors declare that the research was conducted in the absence of any commercial or financial relationships that could be construed as a potential conflict of interest.

## Publisher’s note

All claims expressed in this article are solely those of the authors and do not necessarily represent those of their affiliated organizations, or those of the publisher, the editors and the reviewers. Any product that may be evaluated in this article, or claim that may be made by its manufacturer, is not guaranteed or endorsed by the publisher.
